# Supplementary material for: Sex differences in the development and expression of a preference for familiar vocal signals in songbirds
Source: PLoS One. 2021 Jan 20;16(1):e0243811. doi: 10.1371/journal.pone.0243811 (PMC7816980; doi:10.1371/journal.pone.0243811)
Supplement: S1 Text — (DOCX) [file pone.0243811.s004.docx]

**S4 Text. Additional analysis of song-selective approach responses**

Purpose of analysis

We primarily analyzed the behavioral responses to song playbacks by fitting a statistical model separately to male and female data and compared the results of model estimation between sexes. We chose to analyze all the phonotaxis, calling, and singing responses in this way, assuming that sex differences especially in vocal response may be qualitative rather than quantitative. Regarding the phonotaxis, however, both male and female birds generally showed more approaches towards father’s song, although the decrease in response selectivity was observed in males as they became adult. Thus, we also performed a complementary analysis on phonotaxis where sex was treated as an explanatory variable, so that the possible effect of sex and age on the degree of response selectivity was examined in a different way from the main analysis.

Statistical method

For this purpose, we used a linear mixed model in which the response proportion (time spent in the approach zone of father’s song side divided by the total time spent in both sides, as in Fig. 2) was the response variable. The data acquired at 60 and 120 days post hatch (dph) were used in the same way as in the main analysis. Because one bird of each sex did not visit either approach zones in the test at 60 dph (Table 1), the data of these two birds at 60 dph were excluded from the analysis. The explanatory variables were Sex (male and female were binary coded as 0 and 1, respectively), Age (60 and 120 dph were coded as 0 and 1, respectively), and the interaction of Sex and Age. We assigned subject identity as a random effect. The model was fitted by restricted maximum likelihood estimation. Satterthwaite’s method was used to estimate denominator degree of freedom to compute *t*-value for the coefficients. We conducted model estimation using lme4 package [1] and lmerTest package [2] written in R version 4.0.2 [3].

Results

The result of model estimation is shown in the following table. The estimated coefficients of explanatory variables Sex and Age were relatively small with large standard errors, while the coefficient of interaction was a negative value and significantly different from zero (*p* < 0.05). This is consistent with the result of main analysis that the interaction of stimulus and age contributed to explain the approach response in males but not in females. These analyses collectively indicate that high selectivity of approach to father’s song in female Bengalese finches is maintained after sexual maturation while it slightly decreases in males.

| Term | Estimate | *S.E.* | *df* | *t*-value | *p*-value |
| --- | --- | --- | --- | --- | --- |
| (Intercept) | 0.853 | 0.068 | 32.874 | 12.478 | < 0.001 |
| Sex | 0.036 | 0.097 | 32.874 | 0.370 | 0.713 |
| Age | 0.062 | 0.093 | 17.308 | 0.667 | 0.514 |
| Sex×Age | -0.310 | 0.130 | 16.786 | -2.388 | 0.029 |

References

1. Bates D, Mächler M, Bolker BM, Walker SC. Fitting linear mixed-effects models using lme4. *J Stat Softw*. 2015;67(1):1-48.
2. Kuznetsova A, Brockhoff PB, Christensen RHB. lmerTest package: Tests in linear mixed effects models. *J Stat Softw*. 2017;82(13):1–26.
3. R Core Team. R: A language and environment for statistical computing. R Foundation for Statistical Computing. 2018; https://www.R-project.org/
