# Supplementary material for: Sex differences in the development and expression of a preference for familiar vocal signals in songbirds
Source: PLoS One. 2021 Jan 20;16(1):e0243811. doi: 10.1371/journal.pone.0243811 (PMC7816980; doi:10.1371/journal.pone.0243811)
Supplement: Attachment — Submitted filename: Response to Reviewers 2.docx [file pone.0243811.s006.docx]

We appreciate the reviewers’ and editor’s careful reading and thoughtful comments on our manuscript. We now conducted a complementary analysis on the sex difference of approach behavior as suggested by the editor and reviewer. We also revised the manuscript to clarify some points raised by the reviewer. Specific responses and details about modification are described below (line numbers refer to the latest version of our revised manuscript). We hope that we have correctly addressed all your suggestions, and the analysis will be easier to understand with the supplement and modification.

**Editor:**

*> Your Manuscript is much improved following the revision and both referees commend the efforts. To make the MS still better and more interesting, I would ask for you to consider the complementary analyses suggested.*

Thank you for your advice. We now performed the complementary analysis according to the comment by reviewer 2.

**Reviewer #1:**

*>I am happy with the thorough revision the authors have carried out and with the new manuscript submitted.*

We thank you very much for your comments which improved the manuscript.

**Reviewer #2:**

*>The authors have substantially revised the manuscript and have addressed most points either as suggested. The methods are better to understand now and I have but a few follow up questions and suggestions:*

Thank you for your thorough reading of our revised manuscripts. We additionally modified the manuscript particularly regarding the referencing in the Introduction section and the analysis of sex differences as you pointed out.

*1) Analyses of sex differences: this is the main aim of the study and I understand the authors argumentation to run separate models for males and females where response variables are not fully congruent or because they want to explore which behaviour best predicts their response. However, given that birds generally show individual variation and this is a relatively small sample it is adamant that males and females are also tested in a global model for those parameters that could be measured in both sexes, for example the approach time to familiar/unfamiliar song. You see in both the f/m analyses that there is more approach to the familiar stimulus, I do not see why you cannot, as a posthoc analysis now test whether males and females differ (response time is the dependent, sex*age and their interaction as factors? I think such an analysis is crucial to many of your discussion points?*

*2) Line 243ff Time spent for familiar versus unfamiliar song are mutually exclusive (the time spent in one zone cannot spent be in another). It should therefore be analysed as proportion response time familiar/total response time in approach zones F +UF. This also could simplify your analyses as you have one instead of two response variables.*

Thank you again, for an important suggestion. Regarding these two main comments, we understood your concern that data of males and females should be treated in a single model in which sex, age, interaction are the independent variables, given the individual variation and relatively small sample size in the current experiment. We also agree that approach response (time duration) is comparable between sexes and can be analyzed using the response proportion. Therefore, we performed an additional analysis; a linear mixed model which includes sex, age, and their interaction as explanatory variables was applied to data of response proportion of time spent. We included this analysis in the Supporting Information (S4 Text) and referred to the SI in the main text (lines 244-247, line 667).

*More specific comments by line number*

*> 33 ‘can have’ instead of ‘has’?*

We replaced ‘has’ with ‘can have’ as it was too assertive (line 34).

*> 43 females learn too (as you show here yourself) and see your statement line 46!*

We changed the sentence to correctly convey that only males are ‘vocal’ learners (line 43).

*> 47 m/f learned preference for father’s song has been shown in only a few species; maybe ZF and BF are actually the only ones?*

We now specified that we refer to previous studies that were conducted on zebra finches and Bengalese finches (lines 47-48). We agree that the sentence in previous version told as if it can be generally observed in different species of songbirds.

*> 50 give references for ‘some studies have already demonstrated’ already here?*

We now gave the references here (in this sentence, we intended to summarize the instances cited in the next sentence). (line 51).

*> 55 what do you mean with ‘attached’ here?*

We meant the social attachment (measured by responses such as approaching the tutor). We slightly modified the phrase to more correctly convey this point (line 56).

*> 68 what do you mean with strategy in this context?*

We used the word ‘strategy’ to mean the way a bird perform a given task. As described in the next sentence, there are sometimes individual differences in what cues in the stimuli a bird learns to use. We slightly modified the sentence for clarification (line 69).

*> 69 make clear which study is in which species – it reads as if these are all BF studies*

The target of this meta-analysis paper (ref. 28) was auditory discrimination learning in zebra finches. We now made it clear (line 72).

*>135 is this the same song for all subjects or a different song for each subject?*

Different subjects were tested with different unfamiliar song (different ID of unfamiliar singer). We added this information (lines 136-137).

*> 137 chose from how many songs?*

Since a father’s song for one subject was used, in some cases, as an unfamiliar song for an unrelated subject hatched from another family (lines 140-143), the number of unfamiliar song candidates varied among individuals. For this reason, we cannot specify from how many songs we chose the stimulus, but we think that the number of males (11 fathers and 18 other males, 29 in total) used for stimulus recording in the section of ‘Animals’ can substitute for that.

*> 147 Could you maybe provide an example (or give a ref here)? This might greatly help - not all readers will know BF song?*

We now gave a reference for sound spectrograms of the Bengalese finch songs (line 149). Accordingly, the order of references (refs 35 and 36) changed (lines 152, 176, 605-608).

*> 159 peak sound pressure level?*

Peak sound pressure level was adjusted at 70 dB in the experimental environment (line 162). In line 159 in the previous manuscript, we explained the procedure to control for the variability of amplitude in recorded sounds within each singer.

*> 209 usage of ‘trial’ unclear here – do you mean per song playback?*

We replaced ‘trials’ with ‘playbacks’ (line 211).

*> 210-11 Would counting the calls just after the playback (i.e. in the interval until the new song started?) be also informative of their interest in the song/singer of the song? birds might especially call just after the playback finished?*

Thank you for your suggestion. We initially counted the number of calls during song presentation as well as stimulus intervals. As you pointed out, some birds emitted calls after the stimulus offset, which could also be interpreted as a response to the song. However, in the design of current experiment, mean stimulus duration was long enough (approx. 7 seconds) for a bird to respond during presentation. In addition, as the duration of interval was also relatively long (approx. 23 seconds), we thought that counting all the calls emitted during presentation + intervals would rather decrease the sensitivity of measurement. Although counting the calls emitted during presentation plus a given seconds (for example 2-3 seconds?) just after the stimulus offset may be an alternative, the setting of this time window would be arbitrary. For these reasons, we finally decide to count the calls emitted during presentation.

*> 216-17 but this is what you want to find out?*

As we agree that this justification may contradict our purpose to describe how males and female express their song preference, we deleted the sentence (lines 218-219). Ideally, we should also consider if any call type is particularly relevant to male song preference (for example, by examining correlation to the well-established preference indicator such as phonotaxis). To perform that kind of analysis, however, objective categorization of calls is required as a preliminary step. Although we empirically know that male Bengalese finches have several categories of calls, there has been no literatures that we can refer, and establishing steady criteria of classification anew is out of scope. Thus, we did not conduct call type specific analysis in males but agree that this is rather a technical limitation.

*> 260 for all parameters or only for song?*

We now specified that this applies to all parameters (male approach, female approach, male call, female call, and male song). (line 264)

*> 298 why are you here using proportion but not in analyses explained l 243 ff?*

For a visualization / description purpose, we thought that summarizing the frequency / duration data as a proportion would be easier to understand. However, as it is difficult to assume a specific probability distribution underlying proportion data, it is not suitable for statistical analysis (although conventionally used). Rather, we considered that using the actual frequency / duration value is more appropriate.
